# Identification of lncRNA, miRNA and mRNA expression profiles and ceRNA Networks in small cell lung cancer

**DOI:** 10.1186/s12864-023-09306-4

**Published:** 2023-04-25

**Authors:** Chenxi Zhang, Ying Zhou, Bin Zhang, Zhihong Sheng, Nan Sun, Baiyin Yuan, Xiaoyuan Wu

**Affiliations:** 1 Central Laboratory, Nanjing Chest Hospital, Affiliated Nanjing Brain Hospital, Nanjing Medical University, Nanjing, People’s Republic of China; 2 College of Life Science and Health, Biomedical Research Institute, Wuhan University of Science and Technology, Wuhan, 430081 People’s Republic of China

**Keywords:** RNA-sequencing (RNA-Seq), Small cell lung cancer (SCLC), lncRNA, miRNA, mRNA, ceRNA network, TCONS_00020615

## Abstract

**Background:**

Small cell lung cancer (SCLC) is a highly lethal malignant tumor. It accounts for approximately 15% of newly diagnosed lung cancers. Long non-coding RNAs (lncRNAs) can regulate gene expression and contribute to tumorigenesis through interactions with microRNAs (miRNAs). However, there are only a few studies reporting the expression profiles of lncRNAs, miRNAs, and mRNAs in SCLC. Also, the role of differentially expressed lncRNAs, miRNAs, and mRNAs in relation to competitive endogenous RNAs (ceRNA) network in SCLC remain unclear.

**Results:**

In the present study, we first performed next generation sequencing (NGS) with six pairs of SCLC tumors and adjacent non-cancerous tissues obtained from SCLC patients. Overall, 29 lncRNAs, 48 miRNAs, and 510 mRNAs were found to be differentially expressed in SCLC samples (|log_2_[fold change] |> 1; *P* < 0.05). Bioinformatics analysis was performed to predict and construct a lncRNA-miRNA-mRNA ceRNA network, which included 9 lncRNAs, 11 miRNAs, and 392 mRNAs. Four up-regulated lncRNAs and related mRNAs in the ceRNA regulatory pathways were selected and validated by quantitative PCR. In addition, we examined the role of the most upregulated lncRNA, TCONS_00020615, in SCLC cells. We found that TCONS_00020615 may regulate SCLC tumorigenesis through the TCONS_00020615–hsa-miR-26b-5p–TPD52 pathway.

**Conclusions:**

Our study provided the comprehensive analysis of the expression profiles of lncRNAs, miRNAs, and mRNAs of SCLC tumors and adjacent non-cancerous tissues. We constructed the ceRNA networks which may provide new evidence for the underlying regulatory mechanism of SCLC. We also found that the lncRNA TCONS_00020615 may regulate the carcinogenesis of SCLC.

**Supplementary Information:**

The online version contains supplementary material available at 10.1186/s12864-023-09306-4.

## Background

Small-cell lung cancer (SCLC) is one of the most aggressive malignant neuroendocrine (NE) tumors [1], accounting for 15% of newly diagnosed lung cancers. Compared with NSCLC, SCLC has strong invasiveness, rapid progress, early development of widespread metastases, and many gene mutations. However, there is no major driver gene mutation in SCLC, and no targeted drugs can be used [2]; chemotherapy and radiotherapy are still the main treatment methods for SCLC. Despite initial chemosensitivity, nearly all patients relapse with resistant disease within only a few months, resulting in a 5-year survival rate of 5–10% [3]. The treatment for SCLC has not been significantly improved for more than 30 years [4]. Part of the reason for the lack of progress is that SCLC is rarely treated by surgical resection, and, accordingly, scientific research has been hindered by the lack of available tissue samples. Further research for the molecular mechanisms underlying the occurrence and development of SCLC is urgently needed.

Tough incapable of encoding proteins, lncRNAs hold an indispensable role in epigenetics and gene expression regulation [4]. Emerging studies [5] have established that lncRNAs are crucial during tumor progression as oncogenes or tumor suppressor genes. They participate in biological processes including cell growth, anti-apoptosis, migration, and invasion. Hence, research on lncRNAs may hold significant value in understanding tumor development and progression.

With the rapid development of next-generation sequencing (NGS) technology, long non-coding RNAs (lncRNAs), originally considered as junk molecules, have been identified and demonstrated to play pivotal roles in epigenetics and gene expression regulation. Increasing number of studies indicate that many types of cancer are associated with abnormal expression of lncRNAs, which participate in biological processes of tumor initiation and progression, such as cell proliferation, apoptosis, invasion, and chemosensitivity [5, 6]. As a classic molecular mechanism of lncRNA, the lncRNA-miRNA-mRNA competitive endogenous RNAs (ceRNA) regulatory network is implicated in the development of colorectal cancer [7], pancreatic carcinoma [8], prostate cancer [9], lung cancer [10], and others.

The lncRNA MALAT1 is upregulated in NSCLC and has been reported to modulate miR-204/SLUG and miR-124/STAT3 through two different axes to regulate the progression of lung cancer [11, 12]. Another lncRNA, LOC285194, is downregulated in NSCLC and suppresses NSCLC through targeting p53 [13]. In SCLC, the lncRNA TUG1 promotes cell growth and chemoresistance through regulating the expression of LIMK2b via EZH2 [14]. Knockdown of the lncRNA HIF1A-AS2 increases doxorubicin sensitivity of SCLC cells and decreases autophagy [15]. These newly identified lncRNAs and miRNAs with aberrant expression levels can serve as therapeutic targets and potential diagnostic markers for lung cancer. However, except for these findings, little is known about the role of lncRNAs in SCLC.

In the present study, we identified the global expression profiles of lncRNAs, miRNAs, and mRNAs in SCLC tissues and paired adjacent non-cancerous tissues from six SCLC patients. To the best of our knowledge, this is the first study reporting the RNA expression patterns in SCLC tissues via NGS technology. We predicted a ceRNA network with the differentially expressed lncRNAs, miRNAs, and mRNAs based on miRanda and TargetScan databases. Furthermore, we found that the lncRNA TCONS_00020615, which was the most upregulated gene in SCLC tissues, regulated the proliferation and migration of SCLC cells. The TCONS_00020615–hsa-miR-26b-5p–TPD52 axis may play a pivotal role in SCLC tumorigenesis. Taken together, our study may facilitate the description of the underlying molecular mechanisms of SCLC. Our study may also help in identifying more diagnostic markers and developing novel therapeutic strategies for SCLC.

## Methods

### Human SCLC samples and expression profile dataset

Information of patients and specimens have been provided in our previous report [16]. Briefly, the study included 16 SCLC patients (patient 1–16) who underwent surgery and four SCLC patients (patient 17–20) who underwent fibro-bronchoscopy evaluation without chemotherapy or radiotherapy at our hospital between September 2014 and August 2019. For NGS analysis, six paired SCLC and corresponding adjacent normal tissues were randomly selected, numbered as patient1-6. All the 16 pairs (patient 1–16) of SCLC and adjacent noncancerous tissues, and four SCLC samples (patient 17–20) without matched normal tissue, were used for qRT- PCR validation experiments.

### RNA isolation & RNA-Seq library preparation

Total RNA isolation and RNA-Seq library preparation were performed as previously described [16]. Briefly, total RNA was extracted from human SCLC tissues using TRIzol reagent (Life Technologies, Carlsbad, CA). RNA purity, RNA concentration, and RNA integrity were examined with Agilent 2100 bioanalyzer before RNA-Seq library construction. The library construction was performed using the VAHTS Total RNA-seq (H/M/R) Library Prep Kit for Illumina R (Vazyme Biotech, Nanjing, China) according to the kit’s instructions. The samples were then sequenced (150-bp paired-end RNA-Seq reads) on the Illumina Hiseq X10 platform (Illumina, CA, USA).

### Differentially expression analysis

The raw RNA sequencing (lncRNA, miRNA, and mRNA) reads were post-processed and normalized using the trimmed mean of M-values (TMM) method. EdgeR package in R (version 3.4.1) was used to identify the differentially expressed mRNAs, lncRNAs, and miRNAs between each pair of SCLC sample and adjacent normal sample. The cut-off criteria were set as p ≤ 0.05 and |log_2_(fold change) |> 1. The heat map was plotted using the pheatmap function of pheatmap package version 1.0.8.

### lncRNA-miRNA-mRNA network

Interactions between lncRNAs and miRNAs were predicted using the miRcode database [17]. The MiRanda and TargetScan databases were used to retrieve miRNA-targeted mRNAs. Only the mRNAs targeted by miRNAs present in the two databases were used to construct a ceRNA network. A lncRNA-miRNA-mRNA regulatory network was constructed to visualize the interactions using Cytoscape v3.6.1 [18].

### Functional enrichment analysis

Kyoto Encyclopedia of Genes & Genomes (KEGG) enrichment [19–21] and Gene ontology (GO) [22, 23] analyses were employed to elucidate the potential roles of differentially expressed mRNAs in the ceRNA network. The top 20 enriched KEGG pathways and the top 10 GO terms were plotted.

#### Cell lines, cell culture and establishment of stable knockdown cell lines

Human SCLC cells (NCI-H1688) were obtained from the American Type Culture Collection (ATCC, Manassas, VA, USA). Cells were grown in RPMI 1640 medium (Gibco, USA) supplemented with 10% fetal bovine serum (FBS) and 1% penicillin/streptomycin (Gibco, USA). DNA sequences of shRNAs targeting TCONS_00020615 were subcloned into the pLKO.1-TRC lentivirus vector. The sequences of shRNAs against TCONS_00020615 were 5'-CAGCCCACAAATAAACTGGTA-3'; 5′-GTGCAGGTTGCATTTACTTAT-3'; 5′-AACCAACAGCTTTCAAAGTAA-3'; shRNA directed against GFP (target sequence: 5'-GCAAGCTGACCCTGAAGTTCAT-3') was used as the control scrambled shRNA (shCtrl). Cells were infected with lentivirus carrying specific shRNA in the presence of polybrene (8 μg/mL, sigma) for 24 h. The medium was replaced with fresh medium and the cells were cultured for 48 h. Then the cells were screened with selection medium containing 2.5 μg/mL puromycin until there was no live cell left in the mock group.

### Quantitative real-time PCR (qRT-PCR)

Total RNA (1 μg) from each sample was reverse-transcribed to cDNA using HiScript® II Q RT SuperMix for qRT-PCR (Vazyme). Quantitative PCR was performed on a ROCHE LightCycler® 480 instrument (ROCHE, Basel, Switzerland) using AceQ qRT-PCR SYBR Green Master Mix (without ROX) (Vazyme). All the primers were designed with Primer Premier 5.0 (PREMIER Biosoft, Palo Alto, CA). The qRT-PCR primers used in this study are listed in supplementary Table S1. The relative standard curve method (2 − ΔΔCt) was used to determine the relative lncRNAs and mRNAs expression, β-actin was used as the reference.

### Cell proliferation assay

Cell proliferation was monitored using Cell Counting Kit-8 (CCK8, Vazyme) according to the manufacturer’s instructions. For colony formation assay, cells were seeded on 6-well plates at a density of 2,000 cells per well and cultured in RPMI 1640 medium containing 10% FBS for 10–14 days. Cells were rinsed with PBS and fixed with 4% formaldehyde for 15 min, then stained with 0.5% crystal violet for 30 min. Cell colonies were photographed using a digital camera at 5 × magnification and counted.

### Transwell assay

Costar Transwell chambers with 8-μm aperture (Corning, USA) were used in migration assays. The cells (1 × 10^5^) were suspended in 100 μl RPMI 1640 medium without FBS and transferred to the upper Transwell chambers (Becton, Dickinson and Company, USA), and 600 μl RPMI 1640 medium containing 10% FBS was added into the lower chambers. After 24 h of incubation, cells that passed through the membrane were fixed with 4% formaldehyde and stained with 0.5% crystal violet. Stained cells were imaged and counted using a microscope at 100 × magnification.

### Wound healing assay

Cells were seeded on 6-well plates and grown to 90–100% confluency. Then cells were scratched with a 10 μl sterile pipette tip, washed twice with phosphate buffer saline (PBS), and cultured in RPMI 1640 supplemented with 1% FBS. The wound width was imaged at 0 and 48 h after wounding using a microscope (Leica, Wetzlar, Germany) at 100 × magnification.

### Statistical analyses

Data were analyzed and displayed using GraphPad Prism 5. Data are expressed as means ± SEM. Unpaired two tailed Student’s t-test was used for statistical calculations. Statistically significance was defined as *P* < 0.05. Leave-one-out cross-validation was performed with the qRT-PCR results of sixteen paired (case1 to case16) SCLC and normal tissues in Fig. 5. Generalize linear model was used. Receiver operating characteristic (ROC) curve was drawn by the ggplot2 package on the R platform. Area under Curve (AUC) were calculated by the pROC R package [24].

## Results

### Identification of differentially expressed lncRNAs, miRNAs and mRNAs in SCLC tissues

**Fig. 1:**
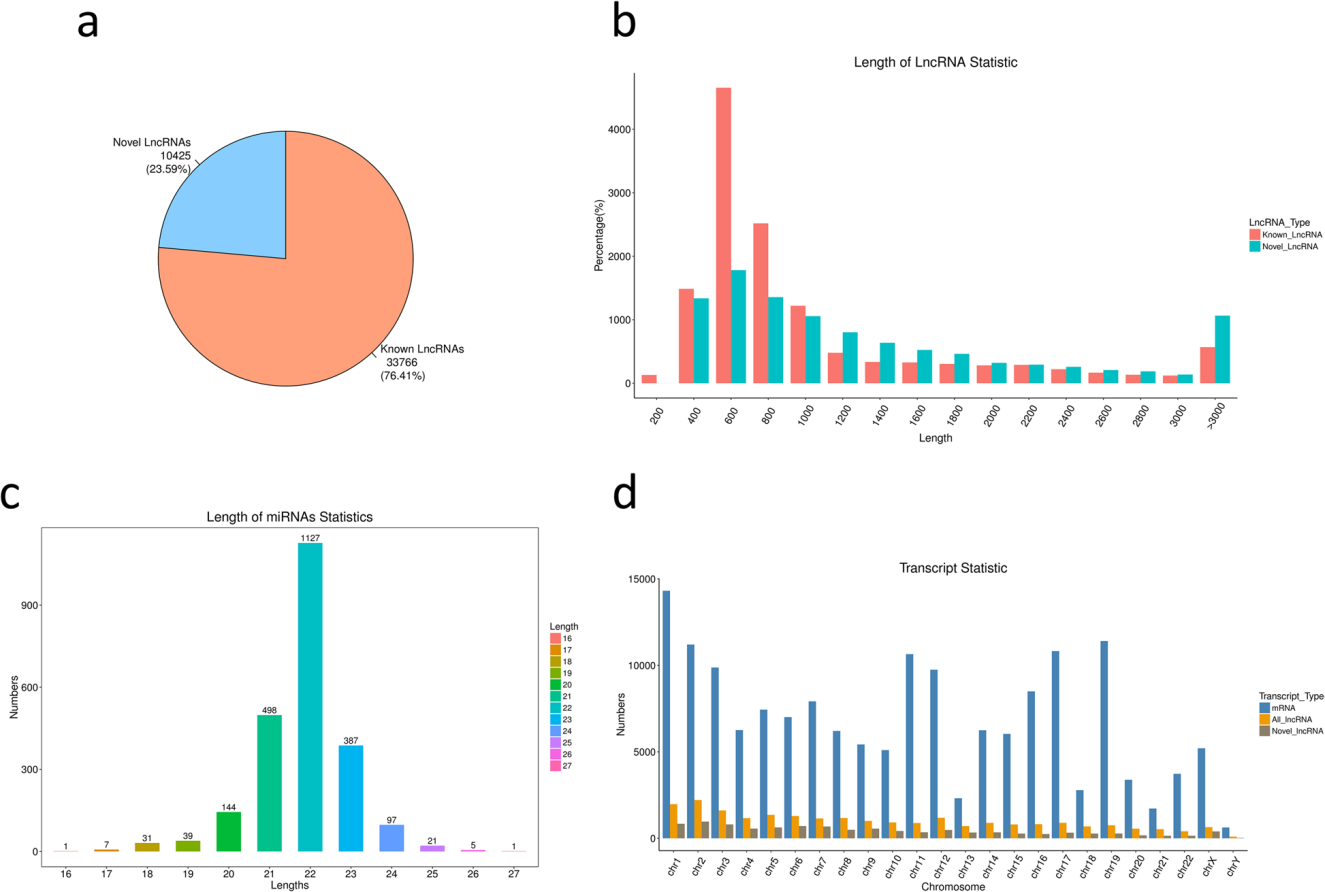
Distribution of lncRNAs, miRNAs, and mRNAs in SCLC tumor tissues and paired adjacent normal tissues. **a**, the lncRNAs identified in this study were compared with previously reported lncRNAs. **b**, length distribution of lncRNAs. **c**, length distribution of miRNAs. **d**, chromosomal distributions of differentially expressed mRNAs and lncRNAs

The NGS technology was utilized to profile lncRNA, miRNA, and mRNA expression in SCLC tumors and adjacent noncancerous tissues from six SCLC patients. In total, 44,191 lncRNAs, 2,358 miRNAs, and 50,869 mRNAs were identified. Compared with previously published studies, 10,425 (23.59%) detected lncRNAs were newly found in this study (Fig. 1a). Most of these detected lncRNAs were 400–1,000 nt in length (Fig. 1b), and most miRNAs consisted of 21–23 nucletides (Fig. 1c). Distributions of lncRNAs and mRNAs in human chromosomes were determined as shown in Fig. 1d; lncRNAs and mRNAs were generally distributed on many chromosomes.

**Fig. 2:**
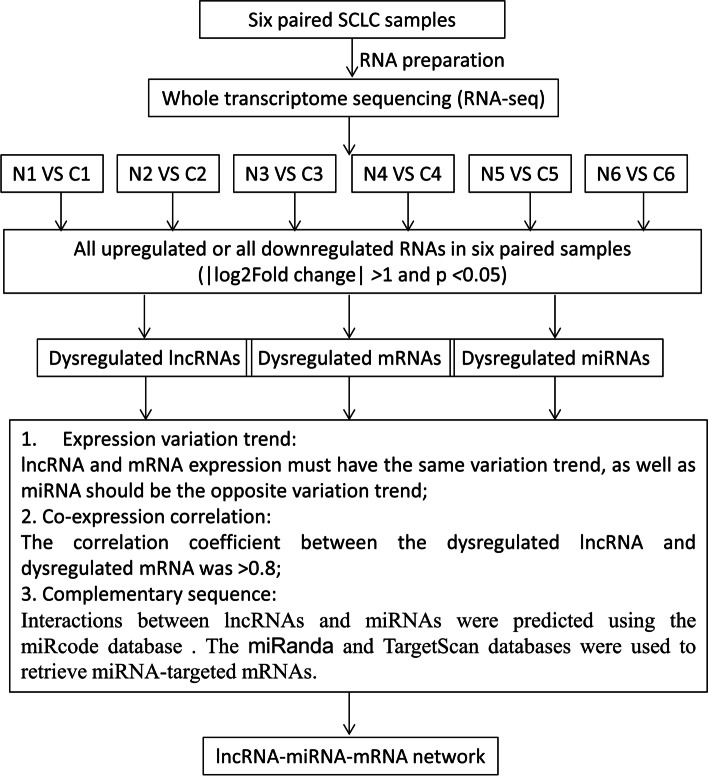
Flow chart of the study design. MRE, miRNA response element

**Fig. 3:**
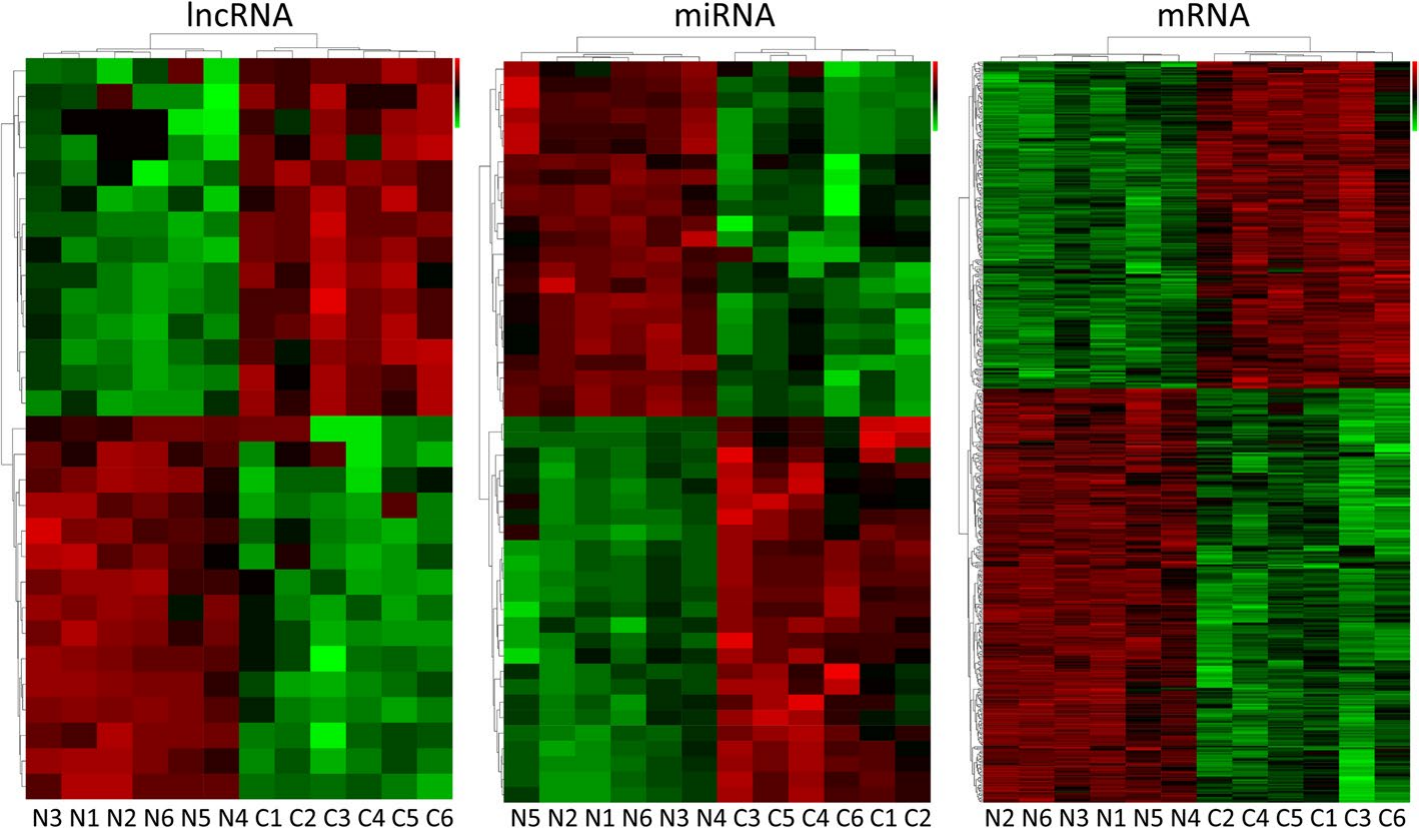
Expression Profiles of lncRNAs, miRNAs, and mRNAs. Heatmap of differently expressed lncRNAs, miRNAs, and mRNAs in the 6 pairs of SCLC tumor tissues and adjacent normal tissues

We compared the expression profiles of lncRNAs, miRNAs, and mRNAs between each pair of SCLC and the corresponding adjacent non-cancerous samples. We selected RNAs with differential expression in all groups using the following cut-off criteria: |log2(Fold Change)|> 1 and *p* < 0.05. The analysis strategy and procedure of the current study are shown in Fig. 2 Overall, 29 lncRNAs (14 upregulated and 15 downregulated), 48 miRNAs (25 upregulated and 23 downregulated), and 510 mRNAs (225 upregulated and 285 downregulated) were dysregulated in the SCLC tissues compared with the non-cancerous tissues. Hierarchical clustering of the identified differentially expressed lncRNAs, miRNAs, and mRNAs were visualized in heatmaps, which exhibited distinguishable characteristic between SCLC tissues and adjacent non-cancerous tissues (Fig. 3).

The top 10 upregulated and downregulated lncRNAs, miRNAs, and mRNAs are listed in Supplementary Table S2. As shown in Table 1, the lncRNA, miRNA, and mRNA with the highest levels of upregulation were TCONS_00223926 (log_2_5.87), hsa-miR-182-3p (log_2_5.12), and CPLX2 (log_2_7.70), respectively; those exhibiting the largest amounts of downregulation were TCONS_00160619 (-log_2_4.826066667), hsa-miR-451a (-log_2_4.99), and SFTPA1 (-log_2_7.36), respectively.

**Table 1:** Statistical analysis of differently expressed lncRNAs, miRNAs, and mRNAs

Differently expressed RNAs	Total No	No. of upregulated	No. of downregulated	The most upregulated (log_2_ Fold Change)	The most downregulated (log_2_ Fold Change)
lncRNA	29	14	15	TCONS_00223926(5.87277)	TCONS_00160619(-4.82607)
miRNA	48	25	23	hsa-miR-182-3p (5.120793545)	hsa-miR-451a(-4.989519163)
mRNA	510	225	285	CPLX2(7.699805)	SFTPA1(-7.360538333)

### Establishment of a ceRNA regulatory network

**Fig. 4:**
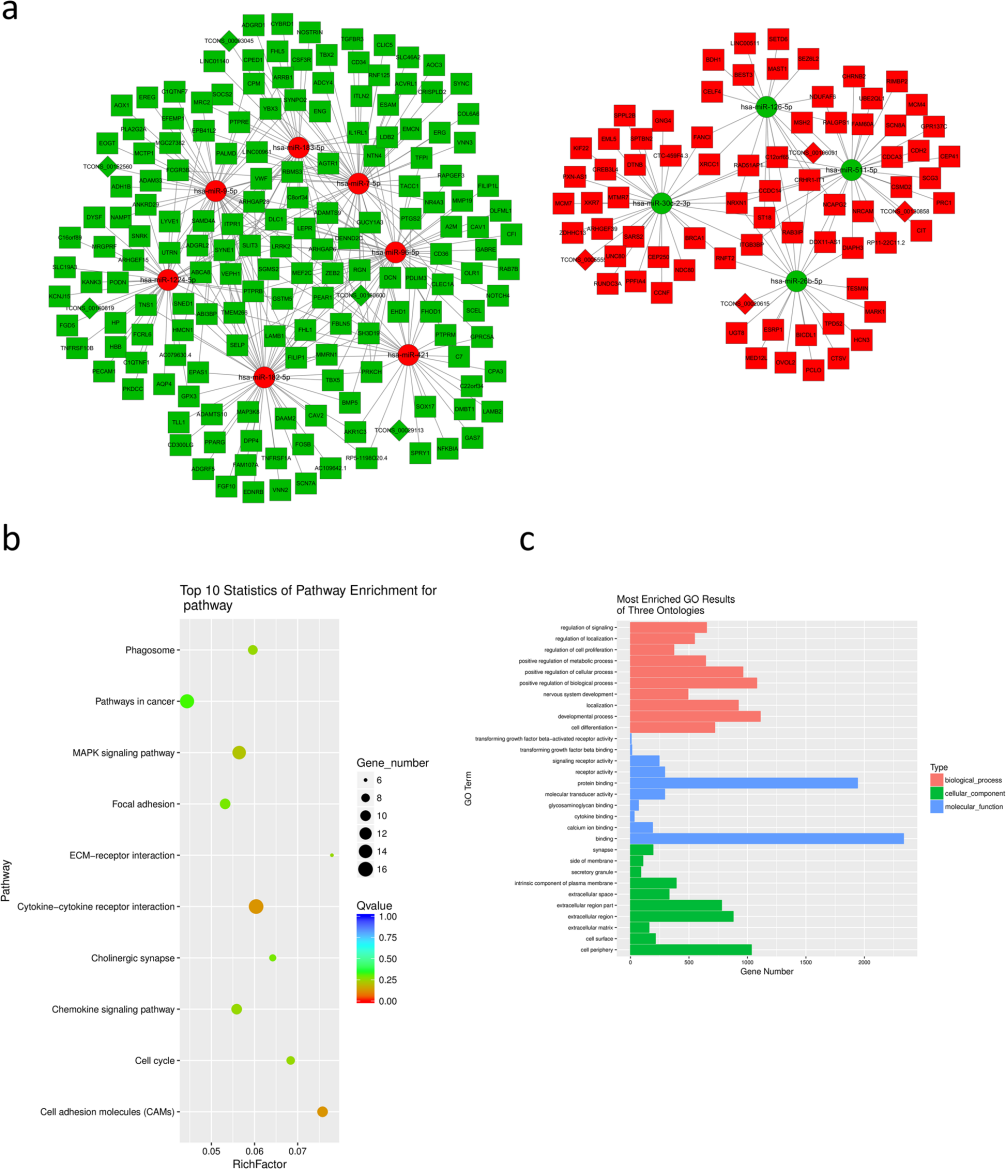
Predicted ceRNA networks based on the RNA expression profiles in SCLC tumors. **a**, lncRNA-miRNA-mRNA network. The nodes highlighted in red indicate upregulation and the nodes highlighted in green indicate downregulation of expression. The RNA species lncRNAs, miRNAs, and mRNAs are represented by diamonds, circles, and squares, respectively. **b**, KEGG pathway and **c**, GO analysis for the mRNAs in the lncRNA-miRNA-mRNAs network

One of the most well-characterized functions of lncRNAs is to act as ceRNAs [25, 26]. To clarify the interaction between these differentially expressed lncRNAs, miRNAs, and mRNAs, we analyzed the lncRNA-miRNA-mRNA regulatory network. The differentially expressed lncRNAs, miRNAs, and mRNAs included in the ceRNA network were filtered as follows. First, we used the miRcode database to predict interactions between lncRNAs with miRNAs. We used miRanda and TargetScan databases to retrieve miRNA-targeted mRNAs. Only miRNA-targeted mRNAs present in both two databases were selected. Second, lncRNAs and mRNAs had to exhibit the same trends of expression, while miRNAs were required to show a trend in the opposite direction. Third, the Pearson correlation coefficient between the differently expressed lncRNAs and mRNAs was required to be greater than 0.8. There were at least three microRNA response elements (MREs) between lncRNAs and miRNAs as well as between miRNAs and mRNAs. As a result, 9 lncRNAs, 11 miRNAs, and 240 mRNAs were selected and used to generate the ceRNA network (Fig. 4a).

### Functional enrichment analysis of differentially expressed mRNAs

To figure out the biological significance of altered levels of RNAs, we further analyzed the 240 ceRNA network-relevant mRNAs through GO and KEGG pathway analyses. GO analysis showed that single-organism cellular process, single-multicellular organism process, and multicellular organism development were the top three enriched biological processes. In the analysis of molecular function, the top three terms were binding, protein binding, and molecular transducer activity. According to the cellular component analysis, proteins encoded by these RNAs were mainly located in cell periphery, plasma membrane, and extracellular region (Fig. 4b). KEGG pathway analysis revealed that these mRNAs were mostly enriched in cytokine-cytokine receptor interaction, pathways in cancer, MAPK signaling pathway, cell adhesion molecules (CAMs), and focal adhesion, which may be related to the progression of SCLC (Fig. 4c).

### Validation of the differently expressed lncRNAs and mRNAs

**Fig. 5:**
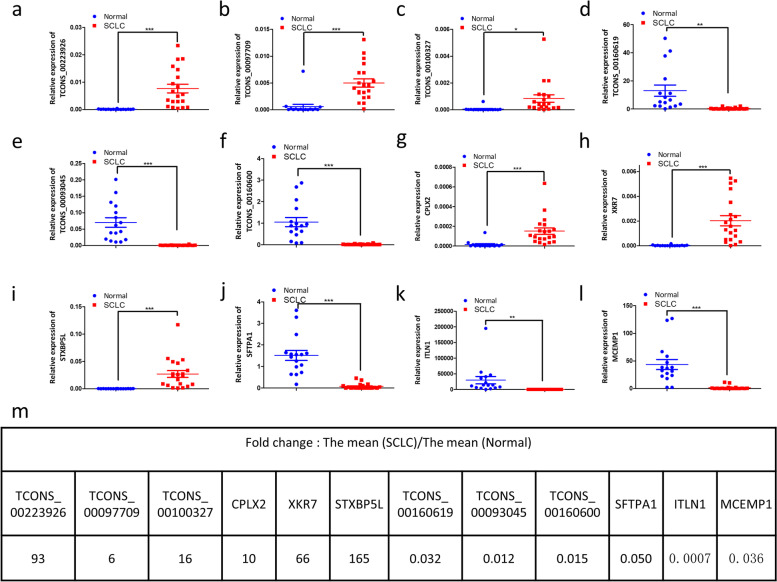
qRT-PCR validation of the top three upregulated and downregulated lncRNAs and mRNAs. **a**-**c**, upregulated lncRNAs. **d**-**f**, downregulated lncRNAs. **g**-**i**, upregulated mRNAs. **j**-**l**, downregulated mRNAs. Expression of all the twelve transcripts had significant changes in SCLC tissues. **m**, expression fold changes of these dysregulated lncRNAs and mRNAs. *, *p* < 0.05; **, *p* < 0.01; ***, *p* < 0.001

To verify the reliability of the RNA-seq data, we firstly examined the expression levels the top three upregulated and downregulated lnRNAs (TCONS_00223926, TCONS_00097709, TCONS_00100327, TCONS_00160619, TCONS_00093045, TCONS_00160600) and mRNAs (CPLX2, XKR7, STXBP5L, SFTPA1, ITLN1, MCEMP1) with qRT-PCR (Fig. 5a- l). qRT-PCR results showed that mRNA levels of all the twelve transcripts had significant changes in SCLC tissues, which were consistent with NGS data. Especially, the upregulated STXBP5L showed 165-fold change and downregulated ITLN1 showed 0.0007-fold change in SCLC tissues (Fig. 5m).

**Fig. 6:**
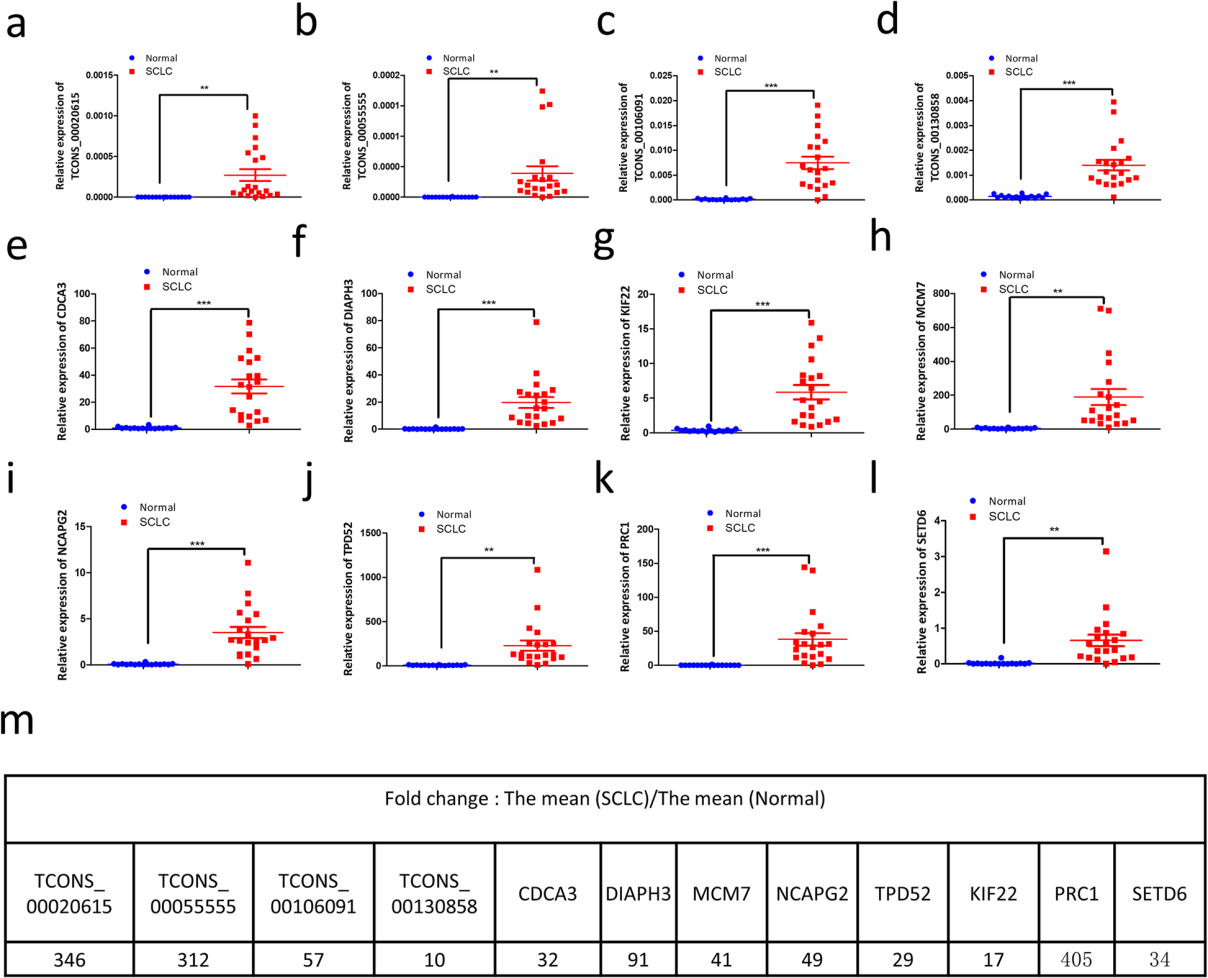
qRT-PCR validation of the dysregulated lncRNAs and mRNAs in the predicted ceRNA network. **a**-**d**, lncRNAs TCONS_00020615, TCONS_00055555, TCONS_00106091, and TCONS_00130858. **e**-**l**, cancer-related mRNAs CDCA3, DIAPH3, MCM7, NCAPG2, TPD52, KIF22, PRC1, and SETD6 were all significantly upregulated in SCLC tissues. **m**, levels of all differently expressed lncRNAs and mRNAs. RNA expression levels were normalized to those of β-actin. **, *p* < 0.01; ***, *p* < 0.001

To identify potential therapeutic targets and molecular mechanisms for SCLC, we also examined the expression levels of the lncRNAs and mRNAs involved in the ceRNA network. Four up-regulated lnRNAs and mRNAs related to them were selected for further research. qRT-PCR results showed that the expression levels of the four lncRNAs, including TCONS_00020615, TCONS_00055555, TCONS_00106091 (also known as LINC00511), and TCONS_00130858, were quiet low in non-cancerous tissues (controls). However, they were significantly upregulated in the corresponding SCLC tissues, especially TCONS_00020615 and TCONS_00055555, which were up-regulated 346-fold and 311-fold in the SCLC tissues, respectively (Fig. 6a–d, k). We selected eight cancer-related mRNAs, including CDCA3 [27], DIAPH3 [28], MCM7 [29], NCAPG2 [30, 31], TPD52 [32], PRC1 [33], SETD6 [34] and KIF22 [35], for qRT-PCR examination. Consistent with the RNA-seq results, mRNA levels of all the eight genes showed significant increases in SCLC tissues (Fig. 6e–l). Fold changes of qRT-PCR validated lncRNAs and mRNAs expression are shown in Fig. 6m. PRC1 exhibited the highest level of upregulation (405-fold; Fig. 6m). Such notable changes in the expression levels of these RNAs in SCLC tissues indicated they may play essential roles in SCLC progression.

**Fig. 7:**
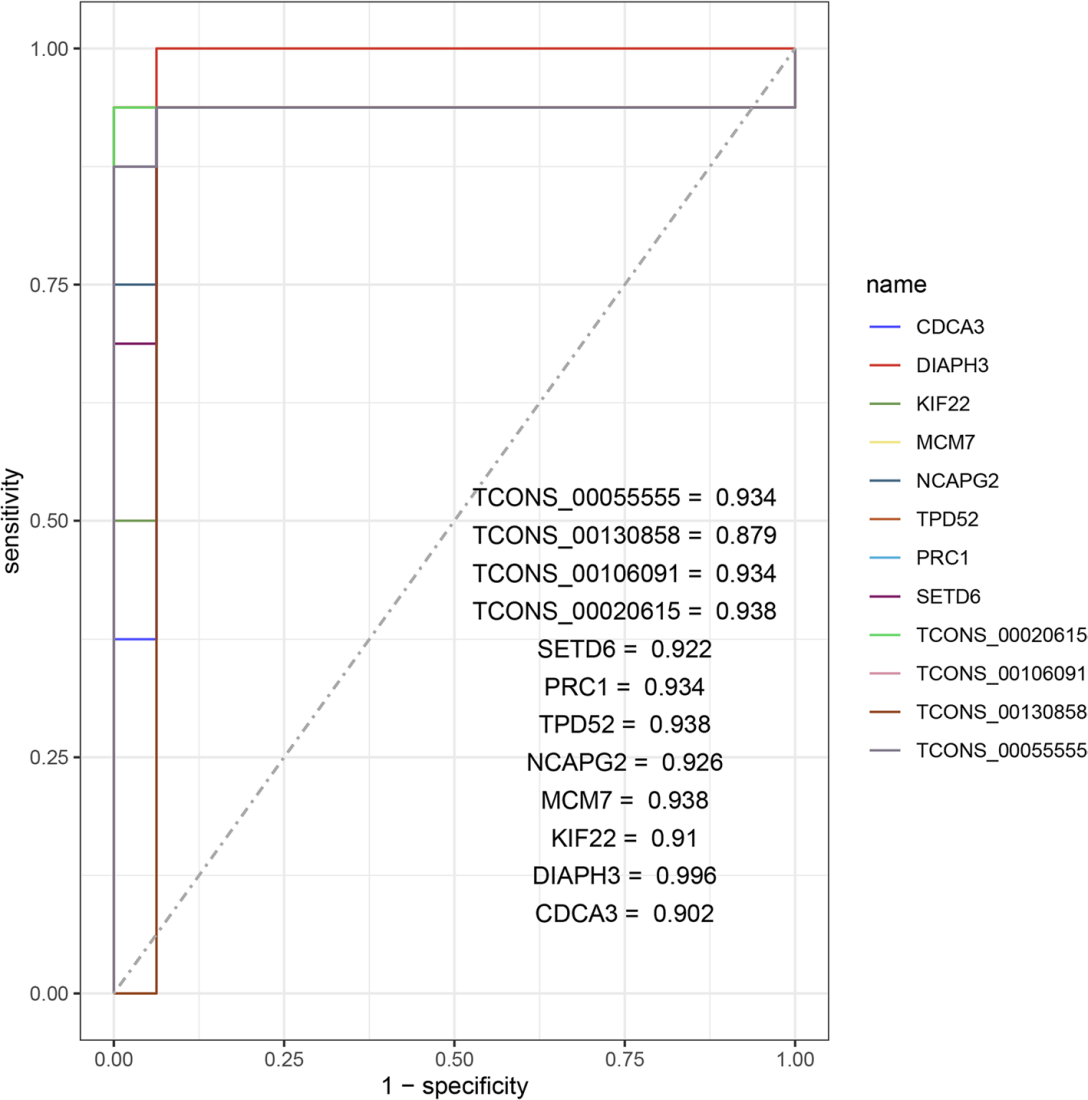
Diagnostic values of the qRT-PCR validated lncRNAs and mRNAs. Leave-one-out cross-validation was performed with the qRT-PCR results of the four lncRNAs and eight mRNAs in the sixteen paired SCLC and normal tissues, and the AUC values of the four lncRNAs and eight mRNAs were shown

To confirm the diagnostic value of these RNAs for SCLC, we performed leave-one-out cross-validation with the qRT-PCR results of the four lncRNAs and eight mRNAs in the sixteen paired (patient 1 to patient 16) SCLC and normal tissues. ROC curves were drawn to evaluate diagnostic efficiency. Results showed that all the twelve RNAs had high accuracy in distinguishing SCLC from the normal tissues (Fig. 7).

### Knock down of TCONS_00020615 hampered the proliferation and migration of SCLC cells

TCONS_00020615 was the lncRNA exhibiting the highest levels of upregulation in SCLC tissues; therefore, we further investigated its potential role in SCLC tumorigenesis. TCONS_00020615 is a 2627-bp-long novel transcript with a sequence complementary to that of PROX1. It is mapped to Chromosome 1: 213,983,793–213,986,419 reverse strand (Supplementary Fig. S1).

**Fig. 8:**
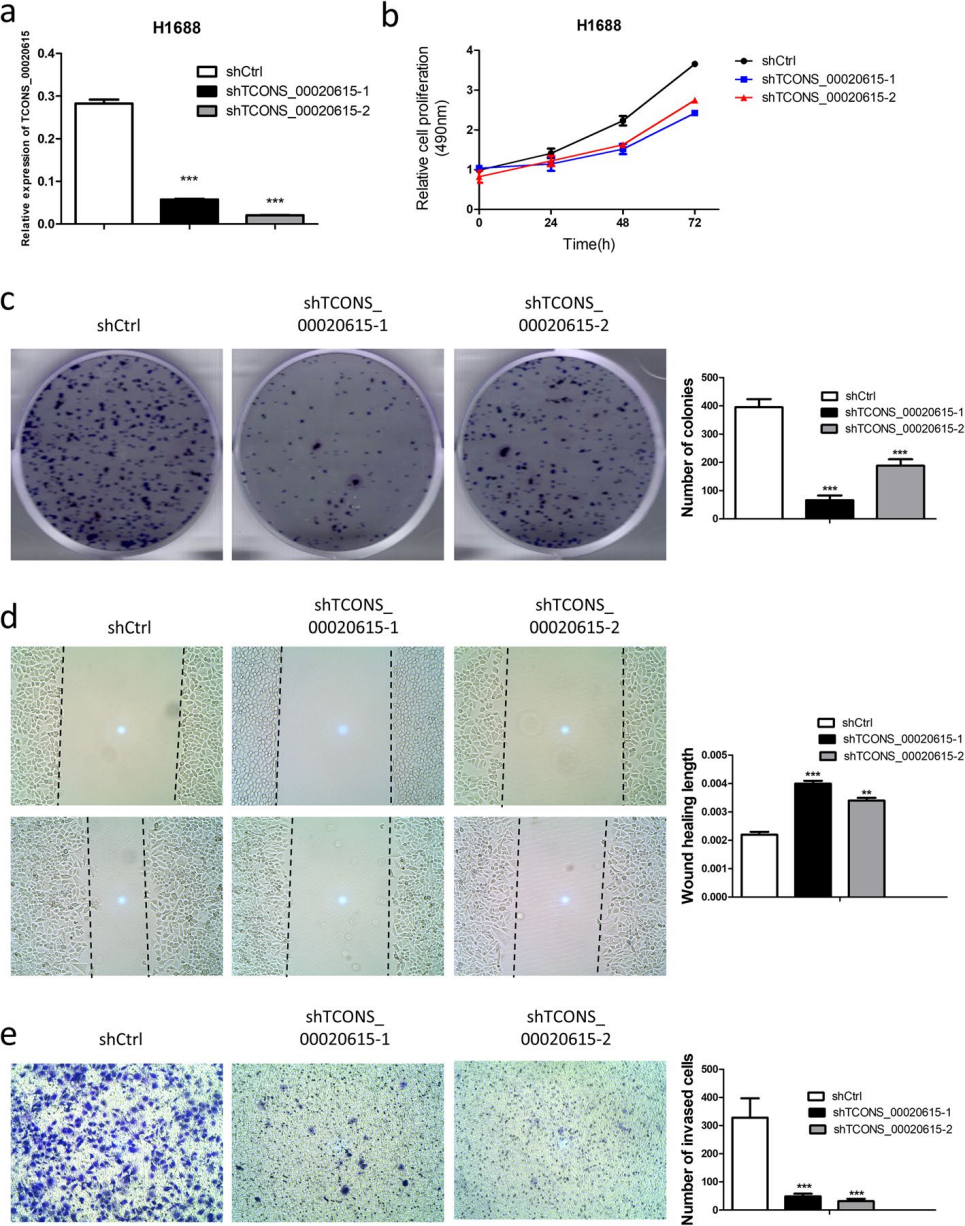
Inhibition of TCONS_00020615 hampers proliferation and migration of SCLC cells. **a**, expression levels of TCONS_00020615 in H1688 cells transfected with shTCONS_00020615. **b**, cell viability as assessed by MTT assay. **c**, colony formation assay and quantification analysis for evaluating cell proliferative ability. **d**, wound healing assay and quantification analysis for monitoring cell migration in H1688 cells. **e**, matrigel invasion assay and quantification analysis for evaluating invasive ability of H1688 cells

To investigate the role of TCONS_00020615 in SCLC tumorigenesis, first, we examined the expression of TCONS_00020615 in human bronchial epithelial cell lines HBE and BEAS-2B as well as in SCLC cell lines H446 and H1688. TCONS_00020615 expression levels in H1688 cells were significantly higher than those in HBE and BEAS-2B cells (Supplementary Fig. S2), so H1688 cells were used for the sequencing studies. Lentivirus-mediated shRNA expression was used to generate a cell line with stable TCONS_00020615 knockdown. Expression of TCONS_00020615 in the knockdown cells were markedly suppressed compared with the control cells as determined by qRT-PCR (Fig. 8a). MTT and colony formation assays were performed in H1688 cells to assess SCLC cell proliferation. TCONS_00020615 knockdown significantly suppressed the proliferation (Fig. 8b) and number of clones (Fig. 8c) in H1688 cells. Wound healing and transwell assays performed to detect cell migratory activity of H1688 cells revealed that TCONS_00020615 knockdown remarkably suppressed the migration and invasion of H1688 cells (Fig. 8d and e). These results suggest that TCONS_00020615 may participate in the SCLC tumorigenesis.

### TCONS_00020615–hsa-miR-26b-5p–TPD52 axis is considered as a potential pathway linked to SCLC

**Fig. 9:**
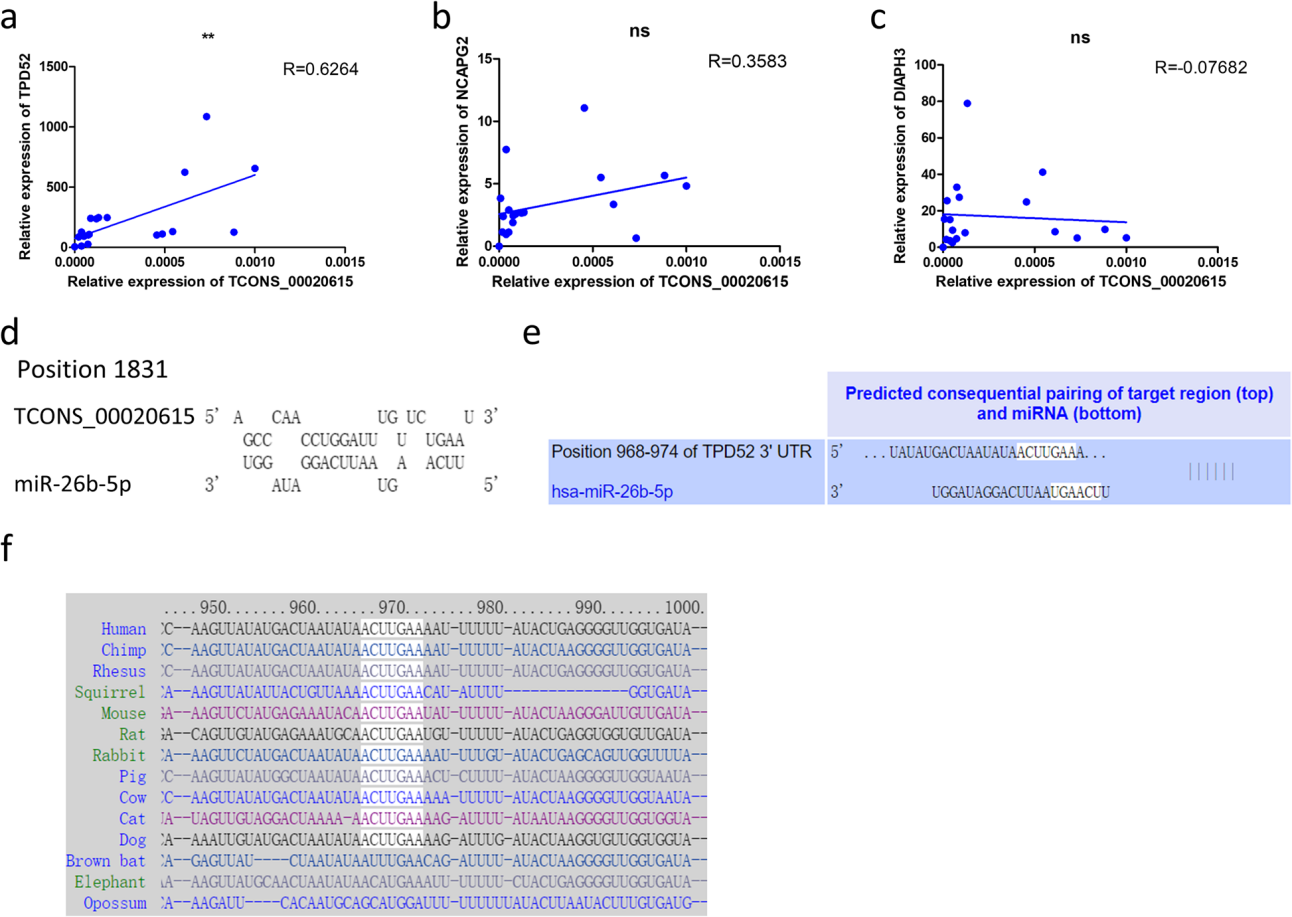
TCONS_00020615–hsa-miR-26b-5p–TPD52 axis is considered as a potential pathway linked to SCLC. **a**-**c**, pearson’s correlation scatter plot of expression levels of TCONS_00020615 and TPD52, NCAPG2, DIAPH3. **d**, potential binding site of TCONS_00020615 and miR-26b-5p. RNAhybrid database (https://bibiserv.cebitec.uni-bielefeld.de/rnahybrid/). **e**, potential binding site of miR-26b-5p and TPD52. f, the potential binding site of miR-26b-5p and TPD52 is broadly conserved among vertebrates. **e** and **f** are the analysis data from Targetscan databse (https://www.targetscan.org/vert_80/)

According to the ceRNA hypothesis, lncRNAs indirectly regulate the expression of mRNAs with a positive correlation. We analyzed the relation between the expression levels of TCONS_00020615 and those of related mRNAs in the constructed ceRNA network. The results indicated that the expression of TCONS_00020615 was positively correlated with that of TPD52 and NCAPG2. However, the p-value for NCAPG2 was not significant (Fig. 9a-c). The miRNA miR-26b-5p was suggested as a target of TCONS_00020615 and an upstream miRNA of TPD52 in the predicted ceRNA network. Results from miRcode database showed that miR-26b-5p had a latent binding site complementary to TCONS_00020615 (Fig. 9d). TargetScan analysis also revealed that TPD52 was considered as a putative target of miR-26b-5p (Fig. 9e). Moreover, the potential binding site of miR-26b-5p and TPD52 is broadly conserved among vertebrates (Fig. 9f). These results indicated that there may be a regulatory relationship between TCONS_00020615 and TPD52.

## Conclusions

To the best of our knowledge, this is the first report providing expression profiles of lncRNAs, miRNAs, and mRNAs in human SCLC tumors and paired adjacent non-cancerous tissues. Comprehensive identification and analyses of the differentially expressed lncRNA-miRNA-mRNA ceRNA regulatory network further indicate potential mechanisms of SCLC progression. The lncRNA with the highest levels of upregulation, TCONS_00020615, may have a role in SCLC tumorigenesis. We anticipate that our work will serve as a valuable resource for future research, which may include diagnosis, mechanistic analyses, and therapy of SCLC.

## Discussion

SCLC is the most malignant subtype of lung cancer [36]. It has two stages: limited stage (LS-SCLC) and extensive stage (ES-SCLC), both of which show the characteristics of fast growth, strong invasiveness, early metastasis, and poor prognosis. Currently, the first-line therapy for SCLC patients is still etoposide plus carboplatin/cisplatin; nearly all ES-SCLC patients relapse with resistant disease within one year, with a median OS of approximately 10–11 months. In recent years, the emergence of immune checkpoint inhibitors has brought new hope for the treatment of ES-SCLC patients [37]. The median OS of SCLC patients treated with anti-PD-L1 mAb is about 12–13 months. Although ICIs plus chemotherapy improved OS of ES-SCLC patients, the therapeutic effect of ICI has not been breakthrough so far [38]. Thus, finding more effective predictive biomarkers and exploring different combination therapy strategies are urgently needed.

Notably, recent studies showed that lung cancer is also associated with dysregulated expression of lncRNAs [10–12]. Unfortunately, because only few tumor specimens can be obtained, information on the genomic profile of SCLC is very limited. In the present study, we performed RNA-Seq to compare the expression profiles of SCLC tumor and adjacent non-cancerous tissues from six SCLC patients. We identified dysregulated RNAs, including 29 lncRNAs, 48 miRNAs, and 510 mRNAs. We also constructed a lncRNA-miRNA-mRNA ceRNA network and finally identified a total of 15 lncRNAs, 17 miRNAs, and 89 mRNAs as potential mediators of SCLC tumorigenesis. We verified the four up-regulated lncRNAs (TCONS_00020615, TCONS_00055555, TCONS_00106091, and TCONS_00130858) and eight cancer-related mRNAs (CDCA3, DIAPH3, MCM7, NCAPG2, TPD52, PRC1, SETD6, and KIF22) involved in the ceRNA network by qRT-PCR. All these lncRNAs and mRNAs exhibited significant upregulation in SCLC tissues.

Among the four up-regulated lnRNAs, TCONS_00020615 and TCONS_00106091 are verified lncRNAs. TCONS_00055555 and TCONS_00130858 are novel lncNRAs that are identified the first time in our sequencing results. TCONS_00106091 (LINC00511) has been studied in a variety of tumors, such as breast cancer [39], gastric cancer [40] and cervical cancer [41]. In NSCLC, two potential LINC00511 targets, enhancer zeste homolog 2 (EZH2) and lysine-specific demethylase 1 (LSD1), have been verified [42, 43]. Expression of LINC00511 was significantly upregulated (57-fold change) in SCLC tissues compared with adjacent non-tumor tissues. However, whether LINC00511 plays an important role in SCLC remains unclear. The role of LINC00511 in SCLC tumorigenesis will be investigated in our future studies.

TCONS_00020615, exhibiting 346-fold higher expression levels in the SCLC tissues, is the most up-regulated lncRNA. TCONS_00020615 is a novel transcript that is antisense to PROX1. Previous studies indicated that lncRNA PROX1-AS1 is up-regulated and acts as a tumor promoter in a wide range of human tumor types, including lung cancer [44], renal cell carcinoma [45], gastric cancer [46] and ovarian cancer [47].. However, expression levels of TCONS_00020615 have not been investigated. In our study, knockdown of TCONS_00020615 reduced the proliferation and migration of HCT1688 cells. Bioinformatic analysis results show that TCONS_00020615 is involved in small cell lung cancer possibly through the TCONS_00020615–hsa-miR-26b-5p–TPD52 pathway.

The miRNA miR-26b-5p has been identified as an oncogenic driver in a variety of cancers, including lymphocytic leukemia [48], hepatocellular carcinoma [49], breast cancer [50], and non-small cell lung cancer (NSCLC) [51, 52]. In NSCLC tissues, miR-26b-5p expression was down-regulated compared with the adjacent non-tumor tissues [51]. Functionally, overexpression of miR-26b-5p suppressed cell proliferation and induces apoptosis in NSCLC by targeting EZH2 [52]. We also observed decreased miR-26b-5p expression in SCLC tissues in our study. There is a putative binding site of miR-26b-5p in TCONS_00020615, suggesting that TCONS_00020615 may exert its tumor-promoting role in SCLC by sponging miR-26b-5p. TPD52 exhibits an increased copy number, and is upregulated in a variety of cancers to accelerate tumor formation and progression by affecting cellular survival, proliferation, migration, invasion, and DNA repair [53].

Eight ceRNA-relevant mRNAs exhibited strinking upregulation in the SCLC samples. Among them, PRC1 showed the highest levels of upregulation (405-fold). Previous studies reported that PRC1 is upregulated in NSCLC tissues and promotes the proliferation and metastasis of NSCLC via activating the Wnt/β-catenin pathway [33]. PRC1 also regulates the tumorigenesis of other types of cancer, including breast cancer [54], uveal melanoma [55], ovarian cancer [56], and childhood cancers, such as Ewing sarcoma (EwS) [57]. DIAPH3 is the secondary upregulated gene (about 100-fold). Previous studies reported that DIAPH3 is also significantly upregulated in lung adenocarcinoma [28], pancreatic cancer [58], and hepatocellular carcinoma [59]. Furthermore, CDCA3, which encodes cell division cycle associated protein-3, is also found to be increased in NSCLC tissues and associated with poor patient prognosis [27]. Zhang et al. reported that CDCA3 is regulated by miR-4677-3p during lung cancer development [60]. Four other genes involved in the ceRNA network, including MCM7 [29], NCAPG2 [31], SETD6 [34], and KIF22 [35] have also been reported to involved in lung cancer progression. These eight genes might be potential therapeutic targets in the small-cell lung cancer, which have not been studied for now.

The limitations of this study can be summarized as follows: First, due to the heterogeneity of SCLC, we adopted relaxed screening conditions and used p-values instead of q-values (corrected p-values) to identify differentially expressed genes. However, it is worth noting that all 24 transcripts selected for qRT-PCR validation exhibited significant changes in our study. Second, the molecular mechanism of TCONS_00020615 in SCLC still needs to be further explored and verified. Third, small sample size and difficulties in obtaining SCLC tissues limit the scope of our study. Further larger scale investigations are needed to analysis the relationship among lncRNAs and clinical features. Nonetheless, to our knowledge, the present study is the first to profile the expression of lncRNA, miRNA, and mRNA, as well as the underlying lncRNA-miRNA-mRNA ceRNA regulatory networks in SCLC. This information could be helpful in improving our understanding of the molecular mechanisms of SCLC and in improving the diagnosis and management of SCLC.

In conclusion, we revealed the expression profiles of lncRNAs, miRNAs, and mRNAs in six pairs of SCLC tumor tissues and adjacent non-cancerous tissues, then we constructed a ceRNA network through the RNA sequencing data. TCONS_00020615 was identified as the most upregulated lncRNA in SCLC patients and might be a potential prognostic marker and therapy target of SCLC. Our findings of these differentially expressed lncRNAs, miRNAs, and mRNAs in the ceRNA network would improve the understanding of the pathogenesis of SCLC and help develop more treatment approaches.

## Supplementary Information

**Additional file 1: Table S1.** The RT-qPCR primer used in this study.** Supplementary Table S2.** The top 10 upregulated and downregulated mRNAs, miRNAs, and lncRNAs.** Supplementary fig S1.** Characteristics of TCONS_00020615. Schematic representation of TCONS_00020615 and PROX1.** Supplementary fig S2.** Relative expression levels of TCONS_00020615 in HBE, BEAS-2B, H1688, and H446 cells.

## Data Availability

Raw sequencing reads of transcriptome Seq of the six pairs of SCLC and corresponding adjacent normal tissues can be obtained from NCBI Sequence Read Archive (SRA) (SRA accession: PRJNA553289). https://www.ncbi.nlm.nih.gov/sra/?term=PRJNA553289

## Abbreviations

SCLC: Small cell lung cancer
lncRNA: Long non-coding RNA
miRNA: MicroRNA
mRNA: Messenger RNA
ceRNA: Competing endogenous RNA
GO: Gene ontology
KEGG: Kyoto encyclopedia of genes and genomes
qRT-PCR: Quantitative real-time PCR
FBS: Fetal bovine serum
PBS: Phosphate bufered saline
CCK8: Cell Counting Kit-8
