# Supplementary material for: Identification of lncRNA, miRNA and mRNA expression profiles and ceRNA Networks in small cell lung cancer
Source: BMC Genomics. 2023 Apr 25;24:217. doi: 10.1186/s12864-023-09306-4 (PMC10131370; doi:10.1186/s12864-023-09306-4)
Supplement: Supplementary file 1 — Additional file 1: Table S1. The RT-qPCR primer used in this study. Supplementary Table S2. The top 10 upregulated and downregulated mRNAs, miRNAs, and lncRNAs. Supplementary fig S1. Characteristics of TCONS_00020615. Schematic representation of TCONS_00020615 and PROX1. Supplementary fig S2. Relative expression levels of TCONS_00020615 in HBE, BEAS-2B, H1688, and H446 cells. [file 12864_2023_9306_MOESM1_ESM.zip › Supplementary data/Supplementary Table S1. The RT-qPCR primer used in this study.docx]

Table S1. The RT-qPCR primer used in this study.

| Name of primer | Sequences |
| --- | --- |
| TCONS_00020615 | F: 5'-AGAAAGAGCGAGCCCGACAG-3'  R: 5'-ATACCTCCAGTGGGACATAGCG-3' |
| TCONS_00055555 | F: 5'-AGAAAGAGCGAGCCCGACAG-3'  R: 5'-ATACCTCCAGTGGGACATAGCG-3' |
| TCONS_00106091 | F: 5'-GCTATCAGGGGTTGTGGC-3'  R: 5'-GCATTTGGACAAGGATGAAC-3' |
| TCONS_00130858 | F: 5'-AAACTAGCAGAAGAATCACCCAC-3'  R: 5'-GGAAATTACACCAGCAGTCAAG-3' |
| TCONS_00223926 | F: 5'-AGGCCTCCTGGATGCTCCTT  ACGCGTGTGTCTGTGTGTGT |
| TCONS_00097709 | F: 5'-TGACACCACCTGGGACCTACA  AGGAGCCACCAAGCTACCCA |
| TCONS_000160619 | F: 5'-GCCCACCCAGGGAGGAAAAC  R: 5'-CAGCCTTCGGAGCTGAGAGC |
| TCONS_00093045 | F: 5'- AGCCTCCAGGTGAGCTGTCA-3'  R: 5'- GGCCCACAGCGTGTTATGGT-3' |
| TCONS_00160600 | F: 5'- CAAAGGCTCACAGCCACCCA-3'  R: 5'- AGGGAGGGAGGCAGGAAGTG-3' |
| TCONS_00100327 | F: 5'- GGGGAAAGCCCCACCTCCTA-3'  R: 5'- CAGGACCCACACAGGAGGGA-3' |
| CPLX2 | F: 5'- GGGTTGGCAACAGAGCAGGT-3'  R: 5'-TGGGAGAGTTCCCACCCAGG-3' |
| XKR7 | F: 5'- GCGCTGCTCGTGTTCTTCT-3'  R: 5'- CGAGTAGTCGTAGACGAACCA-3' |
| STXBP5L | F: 5'- GCTGGAAGTGGTTCCGTACAT-3'  R: 5'- GAACTGGATCAAAGGCTAATGCT-3' |
| SFTPA1 | F: 5'- CCTCTGGTGCTGTGTGCGAA-3'  R: 5'- GCTCCAGGCAGCCCATCATT-3' |
| ITLN1 | F: 5'- ACGTGCCCAATAAGTCCCC-3'  R: 5'- CCGTTGTCAGTCCAACACTTTC-3' |
| MCEMP1 | F: 5'- AGGTGCCCATGACCCAGACT-3'  R: 5'- GCAGAGGACAAAGGCCAGGG-3' |
| NCAPG2 | F: 5'- AAACCGAACATGGCTCAAAAATG-3'  R: 5'-GGCTTCGTAGTTCTCACTTTCA-3' |
| SETD6 | F: 5'-ACCCCGATCTCTTCAGCCTC-3'  R: 5'-CGTTGTGATTGGCTAAGTGGT-3' |
| CDCA3 | F: 5'- CTGGAGGGTCTTAAACATGCC-3'  R: 5'-CACTGCTGGTCTTCATAGGTG-3' |
| PRC1 | F: 5'-ATCACCTTCGGGAAATATGGGA-3'  R: 5'-TCTTTCTGACAGACGGATATGCT-3' |
| DIAPH3 | F: 5'-TGCAAGTAGCTTGTATGCAGC-3'  R: 5'-GGCGATGGGATAACTCAAACA-3' |
| TPD52 | F: 5'- AGCATCTAGCAGAGATCAAGCG-3'  R: 5'-AGCCAACAGACGAAAAAGCAG-3' |
| MCM7 | F: 5'- ACTCTCAGAAACCTACCTGGAAG-3'  R: 5'-CAGCTTTTCGTAGAAATCCTCCT-3' |
| KIF22 | F: 5'-GTTCAGTGCAGCCCATCCTAA-3'  R: 5'-CCAGCATTGTGTGCGTCTT-3' |
| β−actin | F: 5'-GATCATTGCTCCTCCTGAGC-3’  R: 5'-ACTCCTGCTTGCTGATCCAC-3' |
