# Supplementary figures and images for: Identification of lncRNA, miRNA and mRNA expression profiles and ceRNA Networks in small cell lung cancer
Source: BMC Genomics. 2023 Apr 25;24:217. doi: 10.1186/s12864-023-09306-4 (PMC10131370; doi:10.1186/s12864-023-09306-4)

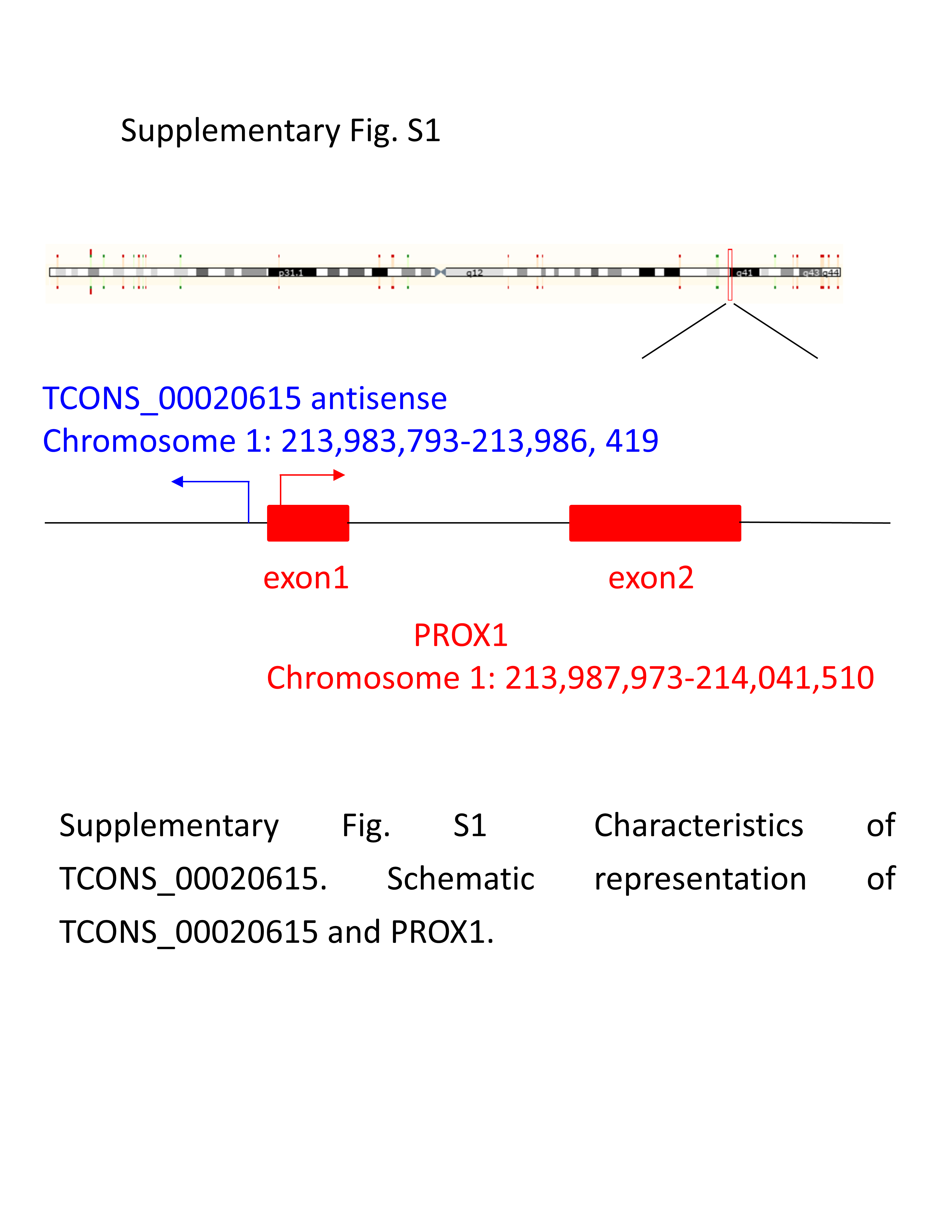

Supplement: Supplementary file 1 — Additional file 1: Table S1. The RT-qPCR primer used in this study. Supplementary Table S2. The top 10 upregulated and downregulated mRNAs, miRNAs, and lncRNAs. Supplementary fig S1. Characteristics of TCONS_00020615. Schematic representation of TCONS_00020615 and PROX1. Supplementary fig S2. Relative expression levels of TCONS_00020615 in HBE, BEAS-2B, H1688, and H446 cells. [file 12864_2023_9306_MOESM1_ESM.zip › Supplementary data/Supplementary Fig. S1 Characteristics of TCONS_00020615.tif]

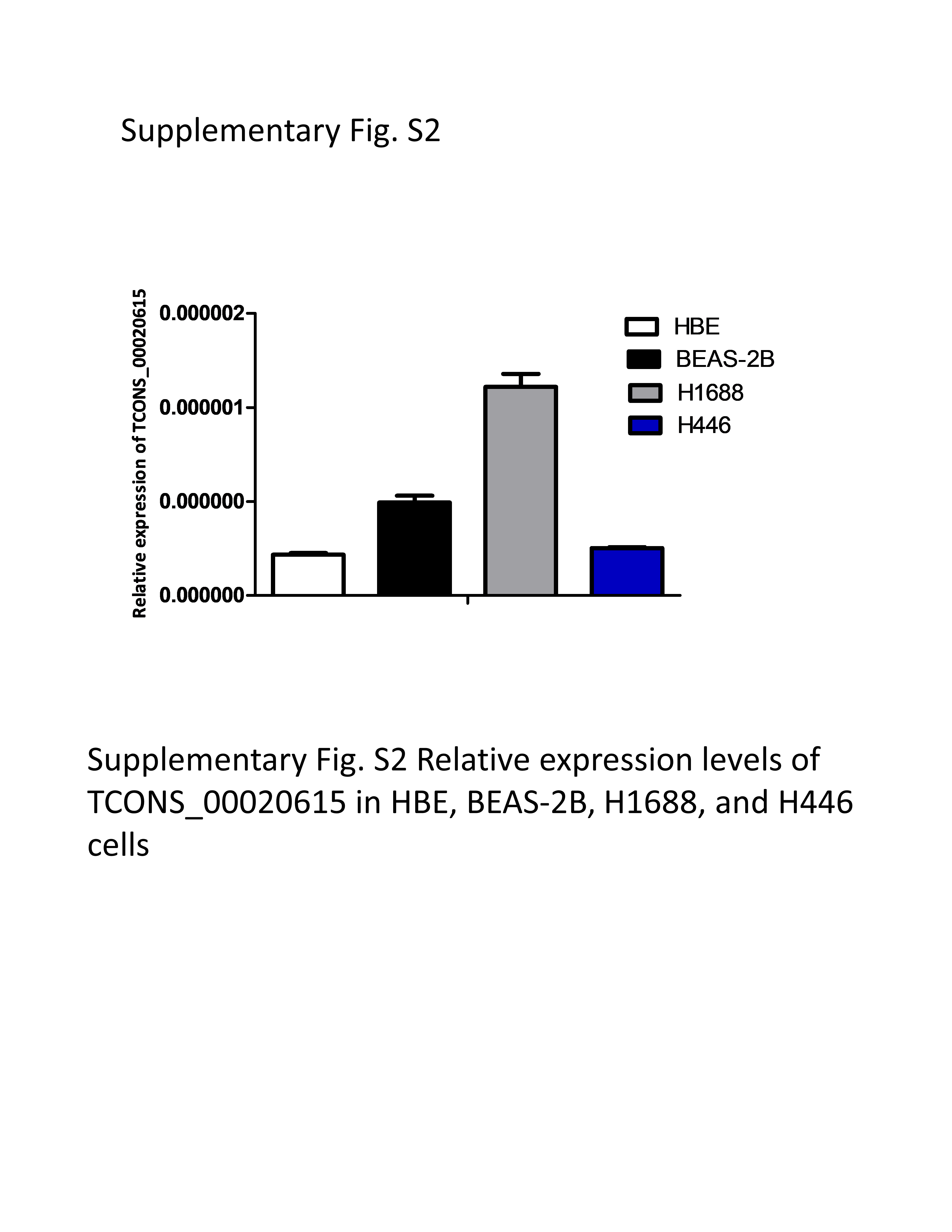

Supplement: Supplementary file 1 — Additional file 1: Table S1. The RT-qPCR primer used in this study. Supplementary Table S2. The top 10 upregulated and downregulated mRNAs, miRNAs, and lncRNAs. Supplementary fig S1. Characteristics of TCONS_00020615. Schematic representation of TCONS_00020615 and PROX1. Supplementary fig S2. Relative expression levels of TCONS_00020615 in HBE, BEAS-2B, H1688, and H446 cells. [file 12864_2023_9306_MOESM1_ESM.zip › Supplementary data/Supplementary Fig. S2 Relative expression levels of TCONS_00020615 in HBE, BEAS-2B, H1688, and H446 cells.tif]
